# Alternating Current Field Effects in Atomically Ferroelectric Ultrathin Films

**DOI:** 10.3390/ma15072506

**Published:** 2022-03-29

**Authors:** Jinming Cao, Mengxia Liu, Zhonglei Liu, Hua Hou, Yuhong Zhao

**Affiliations:** 1 School of Materials Science and Engineering, North University of China, Taiyuan 030051, China; sf190301@st.nuc.edu.cn (J.C.); s1903099@st.nuc.edu.cn (M.L.); s2003070@st.nuc.edu.cn (Z.L.); houhua@nuc.edu.cn (H.H.); 2 School of Materials Science and Engineering, Taiyuan University of Science and Technology, Taiyuan 030024, China

**Keywords:** ferroelectrics, electric field, thin films, domain pattern, phase-field method

## Abstract

In this work, atomically K_1−x_Na_x_NbO_3_ thin films are taken as examples to investigate the reversible and irreversible effects in a horizon plane, i.e., the changes of domain structures, phase states, free energies, etc., under a z-axis alternating current field via a phase-field method. The simulation results show the driving forces during the charging and discharging process, where there is a variation for the angles of the domain walls from 180° to 90° (and then an increase to 135°), which are the external electric field and domain wall evolution, respectively. As for the phase states, there is a transformation between the orthorhombic and rhombohedral phases which can’t be explained by the traditional polarization switching theory. This work provides a reasonable understanding of the alternating current field effect, which is essential in information and energy storage.

## 1. Introduction

As a solid solution of ferroelectric KNbO_3_ and antiferroelectric NaNbO_3_, K_1−x_Na_x_NbO_3_ (KNN) has applications in many fields [1,2], e.g., information encoding [3], energy storage [4], optoelectronic application [5], etc. All applications are closely related to electric fields because they are the origin of polarization [6].

The discovery of KNN began in 1962, when Jona [7] first reported its crystalline, dielectric, piezoelectric, and elastic properties. Three years later, Dungen and Golding [8] explained the polarization phenomenon of KNN, which promoted the vigorous development of KNN.

The mechanism of domain switching under an alternating current field of ferroelectrics has been widely investigated [9], e.g., the electric field influence on the elastic field [10], charged point defects [11], etc. However, the change of ferroelectric domains, e.g., phase states, domain pattern, etc., in a specific plane which is vertical to the direction of the external electric field during the loading and unloading process have rarely been reported, while the current studies have focused more on the domain evolution and properties variations in the same orientation with the electric field, i.e., the z-axis polarization component reverses with the electric field after a certain hysteresis. 

In addition, reducing dimension has become a common and effective way to modify the electric property of materials because of their high sensibility to electric fields [12]. Thus, we choose an atomically KNN thin film as an example to investigate the vertical alternating current (AC) electric field effects in a horizontal plane.

In this work, we describe and explain the domain pattern, phase state, and energy evolution in KNN thin films that are a few atoms thick. In particular, an interesting mechanism is proposed, i.e., a z-axis external electric field that can promote the formation of in-plane vortexes, and, further, to realize ultrahigh data processing and enhance the storage abilities of electric energy [13].

## 2. Methods

The contributions of the domains and domain walls drift are expressed as polarization $P=\left(P_{1},P_{2},P_{3}\right)$ [14]. The domain structures are obtained via the TDGL equation [15]:

(1) $$\frac{\partial P_{i}\left(r,t\right)}{\partial t}=-L\frac{\delta F_{total}}{\delta P_{i}\left(r,t\right)}\;\left(i=1,2,3\right)$$

where $P_{i}\left(r,t\right)$ represents the local polarization vector at the position r and time t and L is the kinetic coefficient. Equation (1) is numerically solved by the semi-implicit Fourier spectral method [16]. $F_{\mathrm{total}}$ [17] is given by:

(2) $$\begin{array}{c} F_{total}=\overset{\;}{\underset{V_{KNN}}{\int }}f_{total}dV \end{array}\;$$

(3) $$\;\;\;\;\;\;\;\;f_{total}=f_{Land}\left(P_{i}\right)+f_{grad}\left(P_{i,j}\right)+f_{elas}\left(P_{i},\varepsilon_{ij}\right)+f_{elec}\left(P_{i},E_{i}\right)$$

where $f_{total}$, $f_{Land}$, $f_{grad}$, $f_{elas}$, and $f_{elec}$ are the local free energy densities of total, Landau, gradient, elastic, and electrostatic energies, respectively, and $V_{\mathrm{KNN}}$ is the volume of the KNN thin film.

$f_{Land}$ can be described as [18]:

(4) $$\begin{array}{l} f_{\mathrm{Land}}\left(P_{i}\right){=\alpha }_{1}\left(P_{1}^{2}{+P}_{2}^{2}{+P}_{3}^{2}\right){+\alpha }_{11}\left(P_{1}^{4}{+P}_{2}^{4}{+P}_{3}^{4}\right){+\alpha }_{12}\left(P_{1}^{2}P_{2}^{2}{+P}_{1}^{2}P_{3}^{2}{+P}_{2}^{2}P_{3}^{2}\right)\\ {+\alpha }_{123}P_{1}^{2}P_{2}^{2}P_{3}^{2}{+\alpha }_{111}\left(P_{1}^{6}{+P}_{2}^{6}{+P}_{3}^{6}\right){+\alpha }_{112}\left(P_{1}^{2}\left(P_{2}^{4}{+P}_{3}^{4}\right){+P}_{2}^{2}\left(P_{1}^{4}{+P}_{3}^{4}\right){+P}_{3}^{2}\left(P_{1}^{4}{+P}_{2}^{4}\right)\right)\\ {+\alpha }_{1111}\left(P_{1}^{8}{+P}_{2}^{8}{+P}_{3}^{8}\right){+\alpha }_{1112}\left(P_{1}^{6}\left(P_{2}^{2}{+P}_{3}^{2}\right){+P}_{2}^{6}\left(P_{1}^{2}{+P}_{3}^{2}\right){+P}_{3}^{6}\left(P_{1}^{2}{+P}_{2}^{2}\right)\right)\\ +\alpha_{1122}(P_{1}^{4}P_{2}^{4}+P_{1}^{4}P_{3}^{4}+P_{2}^{4}P_{3}^{4})+\alpha_{1123}(P_{1}^{4}P_{2}^{2}P_{3}^{2}+P_{1}^{2}P_{2}^{4}P_{3}^{2}+P_{1}^{2}P_{2}^{2}P_{3}^{4}) \end{array}$$

where $\alpha_{1}$, $\alpha_{11}$, $\alpha_{12}$, $\alpha_{123}$, $\alpha_{111}$, $\alpha_{112}$, $\alpha_{1111}$, $\alpha_{1112}$, $\alpha_{1122}$ and $\alpha_{1123}$ are Landau coefficients. The Landau potential coefficients for KNN thin film are listed in Table S1. The contribution of gradient effect $f_{grad}$ can be given as [19]:

(5) $$\begin{array}{c} f_{grad}=\frac{1}{2}G_{11}\left(P_{1,1}^{2}+P_{2,2}^{2}+P_{3,3}^{2}\right)+G_{12}\left(P_{1,1}P_{2,2}+P_{2,2}P_{3,3}+P_{1,1}P_{3,3}\right)\\ +\frac{1}{2}G_{44}\left[{\left(P_{1,2}+P_{2,1}\right)}^{2}+{\left(P_{2,3}+P_{3,2}\right)}^{2}+{\left(P_{1,3}+P_{3,1}\right)}^{2}\right] \end{array}$$

where $G_{11}=0.6\times 10^{-11}\;C^{-2}m^{4}N$, $G_{12}=-0.6\times 10^{-11}\;C^{-2}m^{4}N$, and $G_{44}=0.6\times 10^{-11}\;C^{-2}m^{4}N$ are gradient energy coefficients. In general, the gradient energy density is anisotropic. $f_{elas}$ can be expressed as [20]:

(6) $$\;\;\;\;\;\;\;\;\;\;\;\;\;\;\;\;\;\;\;\;\;\;\;\;\;\;f_{elas}=\frac{1}{2}c_{ijkl}e_{ij}e_{kl}=\frac{1}{2}c_{ijkl}\left(\varepsilon_{ij}-\varepsilon_{ij}^{0}\right)\left(\varepsilon_{kl}-\varepsilon_{kl}^{0}\right),\;\left(i,j,k,l=1,2,3\right)$$

where $c_{ijkl}$, $e_{ij}$, $\varepsilon_{ij}$, and $\varepsilon_{ij}^{0}$ are the elastic stiffness tensor, elastic strain, total strain, and eigenstrain of the KNN thin film, respectively. Here, a mixed-typed elastic boundary condition is employed, i.e., a two-dimensional periodical boundary condition in in-plane directions, while a stress-free boundary condition is in the top and a strain-free boundary condition is in the bottom, which is attached to the substrate, using elastic constants of $c_{11}=2.55848\times 10^{11}\;Pa$, $c_{12}=8.04094\times 10^{10}\;Pa$, and $c_{44}=9.00901\times 10^{10}\;Pa$. The stress-free strain, i.e., eigenstrain $\varepsilon_{ij}^{0}$, caused by the polarization field has the following expression:

(7) $$\left\{ \begin{array}{l} \varepsilon_{11}^{0}\;{=Q}_{11}P_{1}^{2}{+Q}_{12}\left(P_{2}^{2}{+P}_{3}^{2}\right)\\ \varepsilon_{22}^{0}\;{=Q}_{11}P_{2}^{2}{+Q}_{12}\left(P_{1}^{2}{+P}_{3}^{2}\right)\\ \varepsilon_{33}^{0}\;{=Q}_{11}P_{3}^{2}{+Q}_{12}\left(P_{1}^{2}{+P}_{2}^{2}\right)\\ \varepsilon_{23}^{0}\;{=Q}_{44}P_{2}P_{3}\\ \varepsilon_{13}^{0}\;{=Q}_{44}P_{1}P_{3}\\ \varepsilon_{12}^{0}\;{=Q}_{44}P_{1}P_{2} \end{array}\right.$$

where $Q_{11}$, $Q_{12}$, and $Q_{44}$ denote the electrostrictive coefficients. The electrostatic energy density can be described as [21]:

(8) $$f_{elec}=-P_{i}E_{i}-\frac{1}{2}\varepsilon_{0}\kappa_{ij}E_{i}E_{j},\;\left(i,j=1,2,3\right)$$

where $\varepsilon_{0}$ is the vacuum permittivity, $\kappa_{ij}$ denotes the background dielectric constant, and $E_{i}$ represents the component of electric field intensity along an axis. A short-circuit boundary condition is adopted at both the top and bottom surfaces in this work to avoid the depolarization effect, with the assumption that the $\kappa_{ij}$ is isotropic, i.e., $\kappa_{11}{=\kappa }_{22}{=\kappa }_{33}=45$.

In this paper, we use quasi-2D discrete grids of 128∆x×128∆y×40∆z with the grid size ∆x=∆y=∆z=1nm, where the grids of 128∆x×128∆y×10∆z, 128∆x×128∆y×10∆z, and 128∆x×128∆y×20∆z are used to describe the air, thin film, and substrate layers of the system, respectively. 

The initial state of the simulation is the same preset domain structure [22], i.e., the cubic phase, which has a higher energy state to ensure the comparability of calculation results. Considering that, the main purpose of this paper, the electric field dependence, hence the misfit strain, which is constrained by substrates, is set as zero to reduce the influence of the elastic field.

## 3. Results

**Figure 1:**
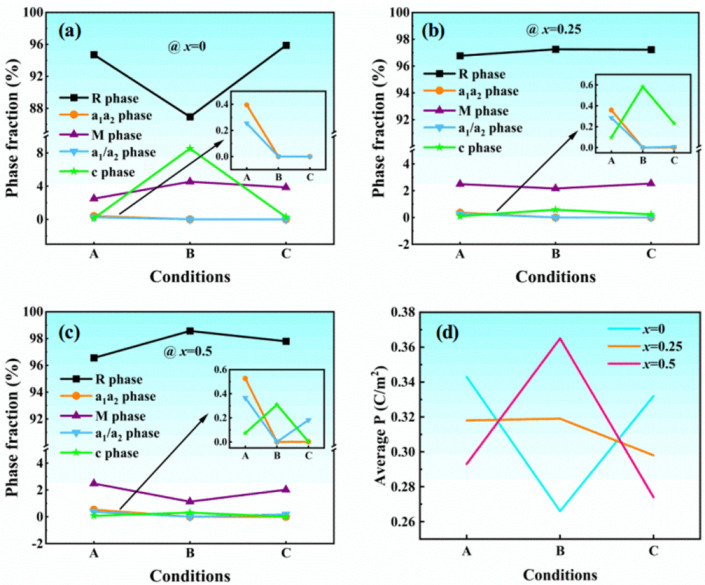
The phase states and average polarization dependence on electric fields. The conditions A, B, and C represent the initial state, loading the maximum electric field, and unloading the electric field, respectively. (**a**) KNbO_3_; (**b**) K_0.75_Na_0.25_NbO_3_; (**c**) K_0.5_Na_0.5_NbO_3_; and (**d**) the average polarization of different conditions.

The phase states and average polarization are shown in Figure 1. Following previous works [23], the obtained domains are classified into seven phases, i.e., R-phase ($P_{1},\;P_{2},\;P_{3}$), $a_{1}a_{2}$-phase ($P_{1},\;P_{2},\;0$), $a_{1}c$-phase ($P_{1},\;0,\;P_{3}$), $a_{2}c$-phase ($0,\;P_{2},\;P_{3}$), $a_{1}$-phase ($P_{1},\;0,\;0$), $a_{2}$-phase ($0,\;P_{2},\;0$), and c-phase ($0,\;0,\;P_{3}$). The $a_{1}c$-phase and $a_{2}c$-phase with the monoclinic symmetry are named as M-phase to distinguish from the $a_{1}a_{2}$-phase, which is called the in-plane phase.

For the A state in Figure 1, we relax the system from a high-energy cubic phase to obtain the equilibrium structure. The results show the domains are filled with R-phase, interspersed with a few M-phase, and very few other phases. This means that the R-phase has lower free energy at room temperature and the polarizations along the z-axis more easily exist. Then, a z-axis 15 V/m electric field, which leads the orientation of the polarization component (the same as the electric field), is loaded gradually to arrive at the B state. Therefore, the R-phase, M-phase, and c-phase would increase while the in-plane phases and $a_{1}/a_{2}$ phases disappear. Finally, we unload the electric field to the C state and there is an obvious trend for the domains to return to their initial state. 

There is a reduction for the average polarization, which then increases in KNbO_3_ while there is a contrast tendency for both K_0.75_Na_0.25_NbO_3_ and K_0.5_Na_0.5_NbO_3_, but the average polarization of all KNN thin films remains lower in the C state than those in the A state. It should be noticed that the tendency of average polarization shows the same with the R-phase. Thus, the preliminary conclusion can be speculated, i.e., maybe the average polarization of the R phase is higher than the others.

The above results indicate that although there are some differences in the changes of the KNN thin films with different Na ratios after loading an alternating current field, these changes are mostly reversible, with the unloading of the electric field, and a few irreversible changes also exist.

## 4. Discussions

Since the electric field applied in this paper is along the z-axis, only the distribution of polarization in the XOY plane needs to be considered. For reasons of this paper’s length, the situations of x=0.25 and x=0 are shown in Figures S1–S4.

**Figure 2:**
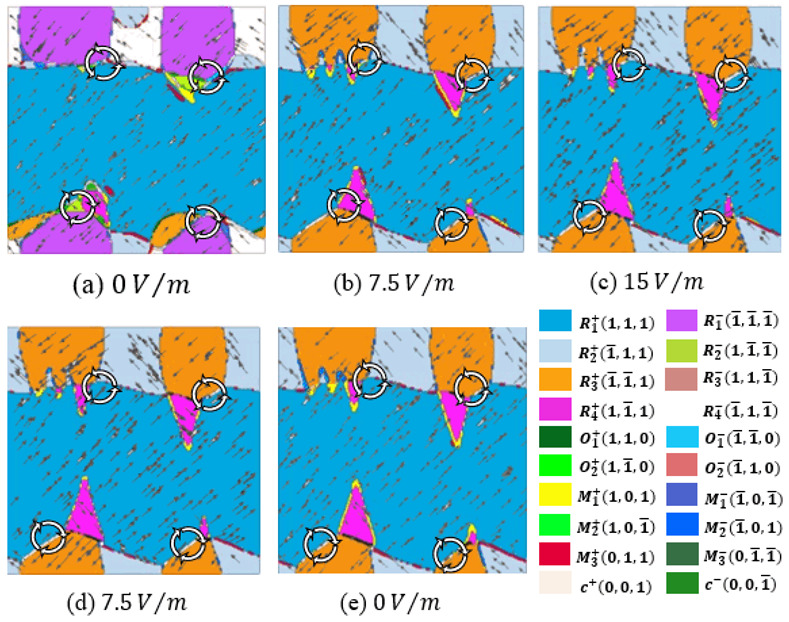
The domain evolution of K_0.5_Na_0.5_NbO_3_ thin films under different electric conditions. (**a**) Without electric field. (**b**,**c**) Loading electric field. (**d**,**e**) Unloading electric field.

Figure 2 shows the domain evolution when loading the electric field. The positions of exotic vortex domains, i.e., the white closed arrow in Figure 2, do not change much, regardless of how the electric field alters. Because of the switching of the z-axis polarization component, the 180° domain walls, e.g., the domain walls between $R_{2}^{+}$ and $R_{4}^{-}$ and $R_{1}^{+}$ and $R_{3}^{-}$, are annihilated, while some $R_{1}^{+}$ and $M_{1}^{+}$ phases transform to $R_{4}^{+}$ phases, which increases the number of domains that make up the vortexes, and thus further stabilize the vortexes.

Subsequently, the external electric field begins to unload, as shown in Figure 2d,e. Although the z-axis electric field does not exist anymore, the domains maintain a positive state. This is the origin of so-called residual polarization.

**Figure 3:**
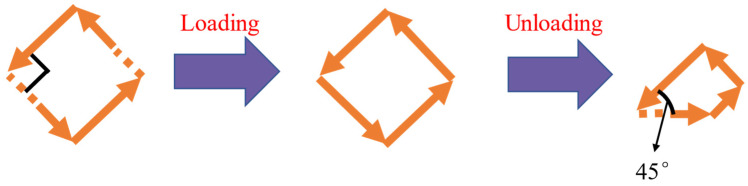
Schematic diagram of vortex variation.

However, the new $R_{4}^{+}$ and $R_{2}^{+}$ phases mentioned in the last paragraph are going to transform into $M_{1}^{+}$ phase, which makes the degree of the new domain walls neither 180°, as in the initial state, nor 90°, as in the quasi-stable state, under the external electric field. Visibly, the morphology of the vortexes has changed dramatically, as shown in Figure 3.

**Figure 4:**
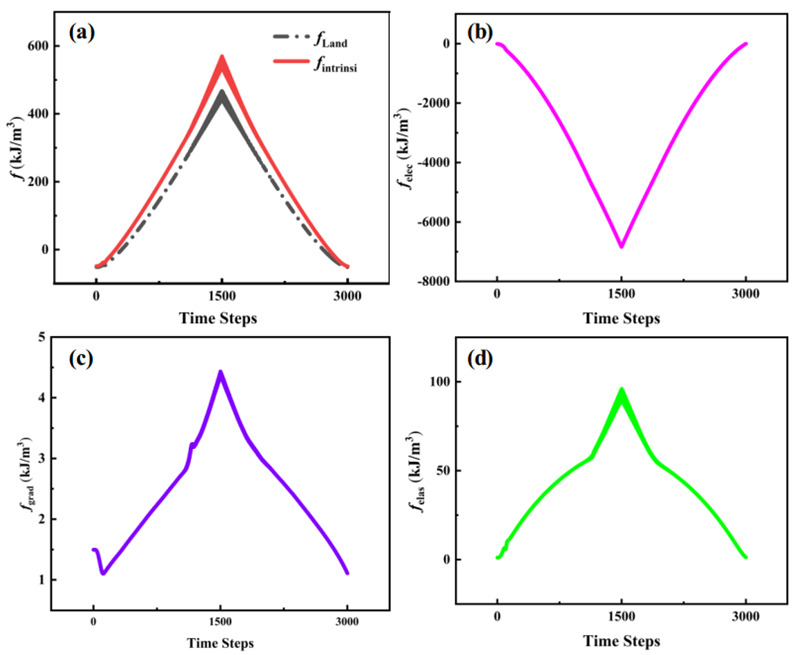
The volumetric average intrinsic energy density (consisting of Landau, gradient, and elastic energy density) and the average electrostatic energy density under different electrical conditions in K_0.5_Na_0.5_NbO_3_ thin films. The time steps of 0–1500 represent the loading process, while the 1500–3000 represent the unloading process. (**a**) Landau and intrinsic energy density. (**b**) Electrostatic energy density. (**c**) Gradient energy density. (**d**) Elastic energy density.

In order to further reveal the relative contribution of the electric field to the domain evolution and phase transition, the different energies for loading and unloading electric fields are systematically studied, as shown in Figure 4. The electric energy $∆f_{electric}=6.8\times 10^{3}\;kJ/m^{3}$ drives the switch of polarizations and maintains the quasi-stable state. There exists an intrinsic energy barrier of $∆f_{barrier}=0.58\times 10^{3}\;kJ/m^{3}$ for switching. During the loading process, there is a decrease of gradient energy at first, which corresponds to the annihilation of the 180° domain walls. Then, it increases to a maximum value which represents the formation of a new domain wall due to the appearance of the $M_{1}^{-}$ phase and $R_{2}^{+}$ phase near the domain walls of the $R_{1}^{-}$ phase, and the presence of the $R_{4}^{+}$ phase near the domain walls of the $R_{1}^{+}$ phase and the $R_{3}^{+}$ phase. There is a small jump in gradient energy density, whose position corresponds to the inflection point in elastic energy density, as seen in Figure 4d. This is because of the disappearance of the $M_{1}^{+}$ phase and the increase of the $R_{4}^{+}$ phase, as seen in Figure 2b. If we unload the electric field, the intrinsic energy would decrease at a high speed, which indicates the previous view, i.e., the existence of irreversible change. The depressed gradient energy and rising electrostatic energy show the annihilation of domain walls and the enhancement of polarization, respectively, which fits well with the results from the domain patterns. Besides, the whole trend of gradient energy indicates that the 180° domain walls have lower energy than the 90° domain walls, which has been proposed by Peng et al. [24]. After the loading of the external electric field, the decrease of gradient energy shows that the domain wall tends to revert to 180° and that the domain wall is the driving force during the loading process. The above conclusions verify the discussion of domain wall angle variation.

## 5. Conclusions

An amazing result shows that an external electric field can alter the direction of polarization in the XOY plane, e.g., the transformation between the $R_{4}^{+}$ and $M_{1}^{+}$ phases. The external electric field is the main driving force during the loading process, while the domain wall motion is the counterpart during the unloading process. There is a negative correlation between the domain wall angle and domain wall energy at the range of 90° to 180° in the XOY plane. 

In particular, several interesting phase transitions between in-plane phases and the R phase or O phase are shown during the loading and unloading process, which are caused by the gradient energy.

Thus, a formal calculation method is constructed to understand the phase transitions during the physical process, and the vortex domains are demonstrated to drive the above transitions. This is what has never been reported in previous work and can be used to modify the energy or information storage properties via a domain project.

## Data Availability

The raw data in this paper and its Supplemental Information Files are available from corresponding author (zhaoyuhong@nuc.edu.cn) or first author (sf190301@st.nuc.edu.cn) upon reasonable request.

## Supplementary Materials

The following supporting information can be downloaded at: https://www.mdpi.com/article/10.3390/ma15072506/s1, Figure S1: The domain evolution of KNbO_3_ thin films under different electric conditions. (a) without electric field; (b, c) loading electric field; (d, e) unloading electric field.; Figure S2: The domain evolution of K_0.75_Na_0.25_NbO_3_ thin films under different electric conditions. (a) without electric field; (b, c) loading electric field; (d, e) unloading electric field.; Figure S3: Dynamic temporal evolution of the volumetric average intrinsic energy density (consisting of Landau, gradient, and elastic energy density) and the average electrostatic energy density under different electrical conditions in KNbO_3_ thin films. The 0–1500 time steps represent the loading process while the 1500–3000 represent the unloading process. (a) Landau and intrinsic energy density. (b) Electrostatic energy density. (c) Gradient energy density. (d) Elastic energy density.; Figure S4: Dynamic temporal evolution of the volumetric average intrinsic energy density (consisting of Landau, gradient, and elastic energy density) and the average electrostatic energy density under different electrical conditions in K_0.75_Na_0.25_NbO_3_ thin films. The 0–1500 time steps represent the loading process while the 1500–3000 represent the unloading process. (a) Landau and intrinsic energy density. (b) Electrostatic energy density. (c) Gradient energy density. (d) Elastic energy density.; Table S1: Related coefficients for K_1−x_Na_x_NbO_3_ crystal.
