# Supplementary material for: Alternating Current Field Effects in Atomically Ferroelectric Ultrathin Films
Source: Materials (Basel). 2022 Mar 29;15(7):2506. doi: 10.3390/ma15072506 (PMC8999760; doi:10.3390/ma15072506)
Supplement: Supplementary file 1 [file materials-15-02506-s001.zip › materials-1618211-supplementary.pdf]

materials

Article

Alternating Current Field Effects in Atomically Ferroelectric Ultrathin Films

Jinming Cao [1], Mengxia Liu [1], Zhonglei Liu [1], Hua Hou [1,2] and Yuhong Zhao [1,*]

1 School of Materials Science and Engineering, North University of China, Taiyuan 030051, China; sf190301@st.nuc.edu.cn (J.C.); s1903099@st.nuc.edu.cn (M.L.); s2003070@st.nuc.edu.cn (Z.L.); houhua@nuc.edu.cn (H.H.)
2 School of Materials Science and Engineering, Taiyuan University of Science and Technology, Taiyuan 030024, China
* Correspondence: zhaoyuhong@nuc.edu.cn

Abstract: In this work, atomically $K_{1\text{-}x}Na_xNbO_3$ thin films are taken as examples to investigate the reversible and irreversible effects in a horizon plane, i.e., the changes of domain structures, phase states, free energies, etc., under a z-axis alternating current field via a phase-field method. The simulation results show the driving forces during the charging and discharging process, where there is a variation for the angles of the domain walls from 180° to 90° (and then an increase to 135°), which are the external electric field and domain wall evolution, respectively. As for the phase states, there is a transformation between the orthorhombic and rhombohedral phases which can't be explained by the traditional polarization switching theory. This work provides a reasonable understanding of the alternating current field effect, which is essential in information and energy storage.

Keywords: ferroelectrics; electric field; thin films; domain pattern; phase-field method

Citation: Cao, J.; Liu, M.; Liu, Z.; Hou, H.; Zhao, Y. Alternating Current Field Effects in Atomically Ferroelectric Ultrathin Films. Materials **2022**, *15*, 2506. https://doi.org/10.3390/ma15072506

Academic Editor: Ioana Pintilie

Received: 14 February 2022
Accepted: 22 March 2022
Published: 29 March 2022

Publisher's Note: MDPI stays neutral with regard to jurisdictional claims in published maps and institutional affiliations.

Copyright: © 2022 by the authors. Licensee MDPI, Basel, Switzerland. This article is an open access article distributed under the terms and conditions of the Creative Commons Attribution (CC BY) license (https:// creativecommons.org/licenses/by/4.0/).

Supplementary Note 1: **Related coefficients**

Table S1. Related coefficients for $K_{1\text{-}x}Na_xNbO_3$ crystal.

Coefficients	Value	Units
α_1	$2x \times 4.2900 \times 10^7 \times \left(coth\frac{140}{T} - coth\frac{140}{657}\right)$ $+(1-2x) \times 5.9822 \times 10^7 \times \left(coth\frac{140}{T} - coth\frac{140}{650}\right)$	$C^{-2}m^2N$
α_{11}	$2x \times (-2.7302) \times 10^8 + (1-2x) \times (-6.36) \times 10^8$	$C^{-4}m^6N$
α_{12}	$2x \times 1.0861 \times 10^9 + (1-2x) \times 9.66 \times 10^8$	$C^{-4}m^6N$
α_{123}	$2x \times 1.5513 \times 10^{10} + (1-2x) \times 4.50 \times 10^9$	$C^{-6}m^{10}N$
α_{111}	$2x \times 3.0448 \times 10^9 + (1-2x) \times 2.81 \times 10^9$	$C^{-6}m^{10}N$
α_{112}	$2x \times (-2.7270) \times 10^9 + (1-2x) \times (-1.99) \times 10^9$	$C^{-6}m^{10}N$
α_{1111}	$2x \times 2.4044 \times 10^{10} + (1-2x) \times 1.74 \times 10^{10}$	$C^{-8}m^{14}N$

Coefficients	Value	Units
α_{1112}	$2x \times 3.7328 \times 10^{9} + (1-2x) \times 5.99 \times 10^{9}$	$C^{-8}m^{14}N$
α_{1122}	$2x \times 3.3485 \times 10^{10} + (1-2x) \times 2.50 \times 10^{10}$	$C^{-8}m^{14}N$
α_{1123}	$2x \times (-6.2017) \times 10^{10} + (1-2x) \times (-1.17) \times 10^{10}$	$C^{-8}m^{14}N$
G_{11}	0.6×10^{-11}	$C^{-2}m^{4}N$
G_{12}	-0.6×10^{-11}	$C^{-2}m^{4}N$
G_{44}	0.6×10^{-11}	$C^{-2}m^{4}N$
c_{11}	2.55848×10^{11}	Pa
c_{12}	8.04094×10^{10}	Pa
c_{44}	9.00901×10^{10}	Pa
$\kappa_{11} = \kappa_{22} = \kappa_{33}$	45	

where the temperature is set as $T = 298$ K.

Supplementary Note 2: **Domain evolution**

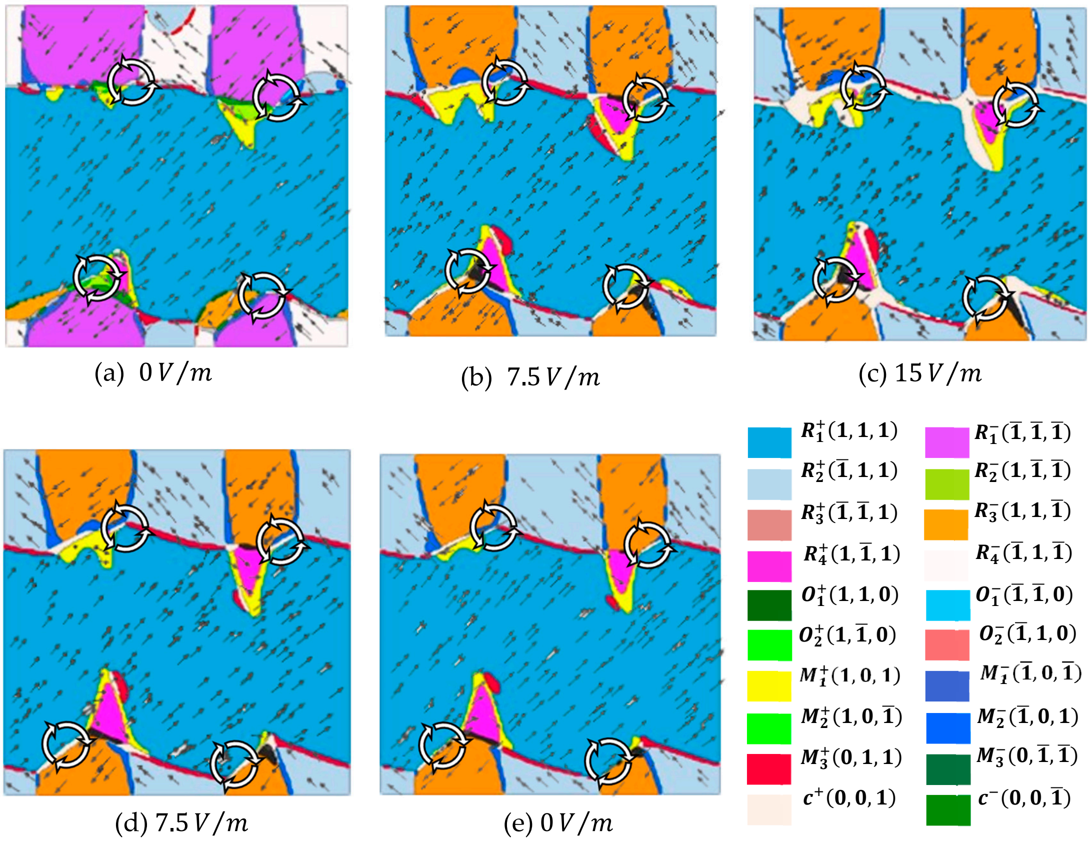

Figure S1. The domain evolution of $KNbO_3$ thin films under different electric conditions. (**a**) without electric field; (**b**,**c**) loading electric field; (**d**,**e**) unloading electric field.

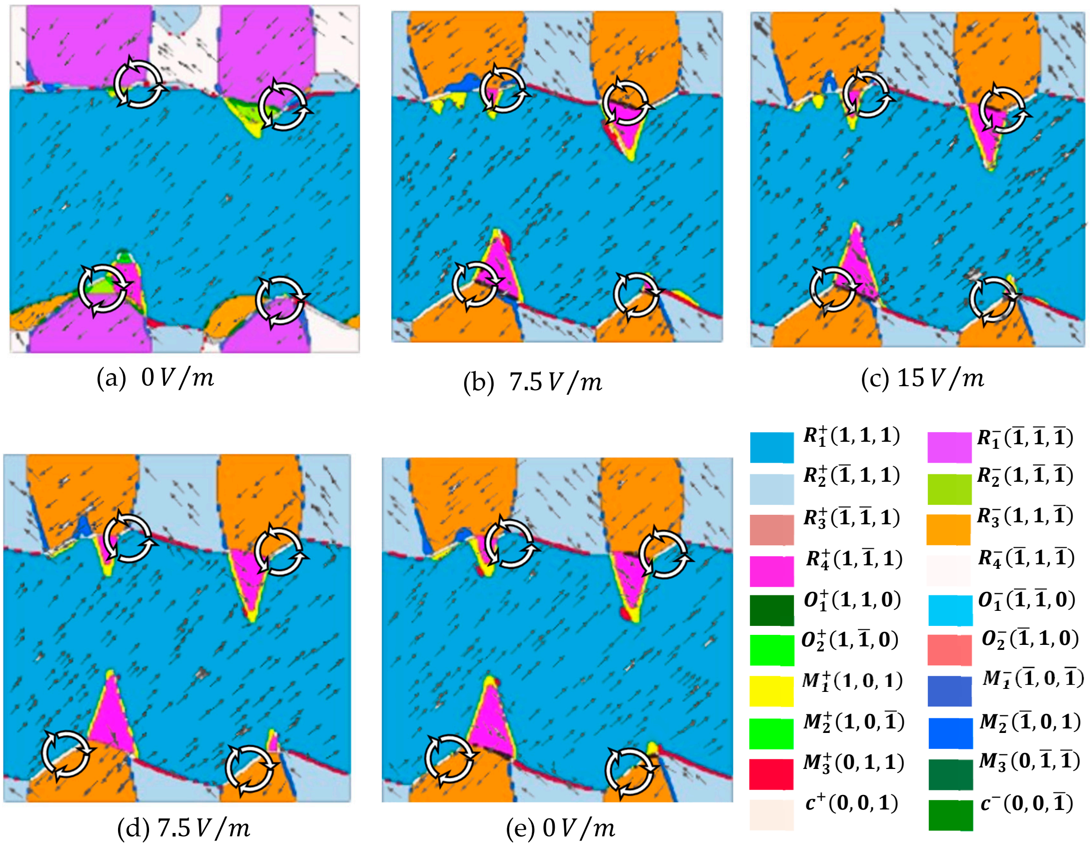

Figure S2. The domain evolution of $K_{0.75}Na_{0.25}NbO_3$ thin films under different electric conditions. (**a**) without electric field; (**b**,**c**) loading electric field; (**d**,**e**) unloading electric field.

Supplementary Note 3: **Free energy density evolution**

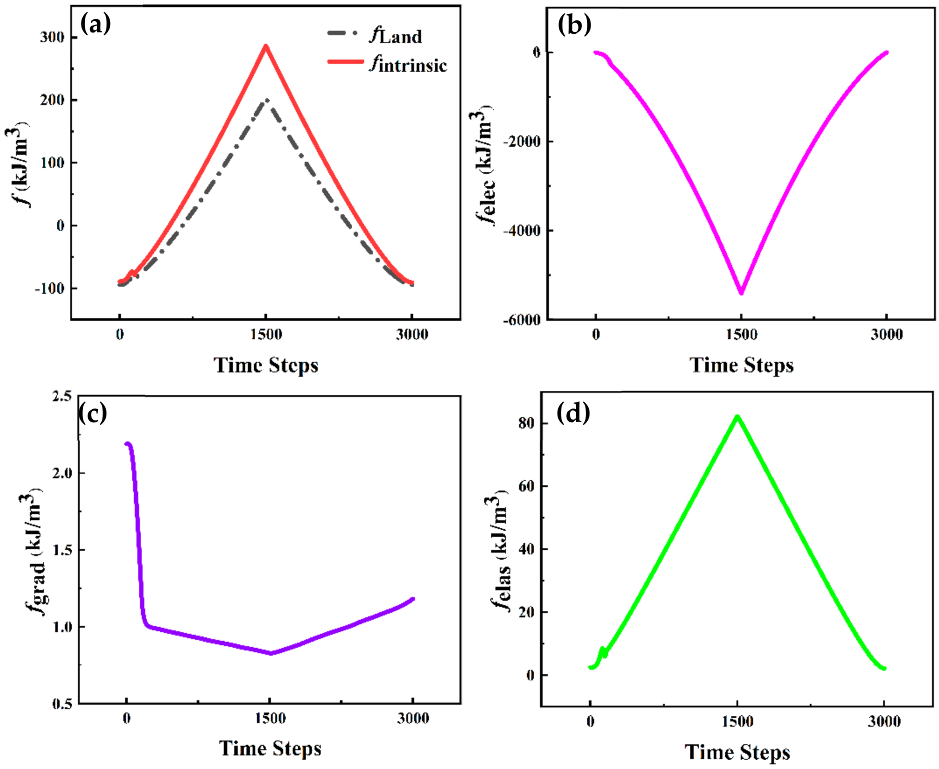

Figure S3. Dynamic temporal evolution of the volumetric average intrinsic energy density (consisting of Landau, gradient, and elastic energy density) and the average electrostatic energy density under different electrical conditions in $KNbO_3$ thin films. The 0–1500 time steps represent the loading process while the 1500–3000 represent the unloading process. (**a**) Landau and intrinsic energy density. **(b)**Electrostatic energy density. (**c**) Gradient energy density. (**d**) Elastic energy density.

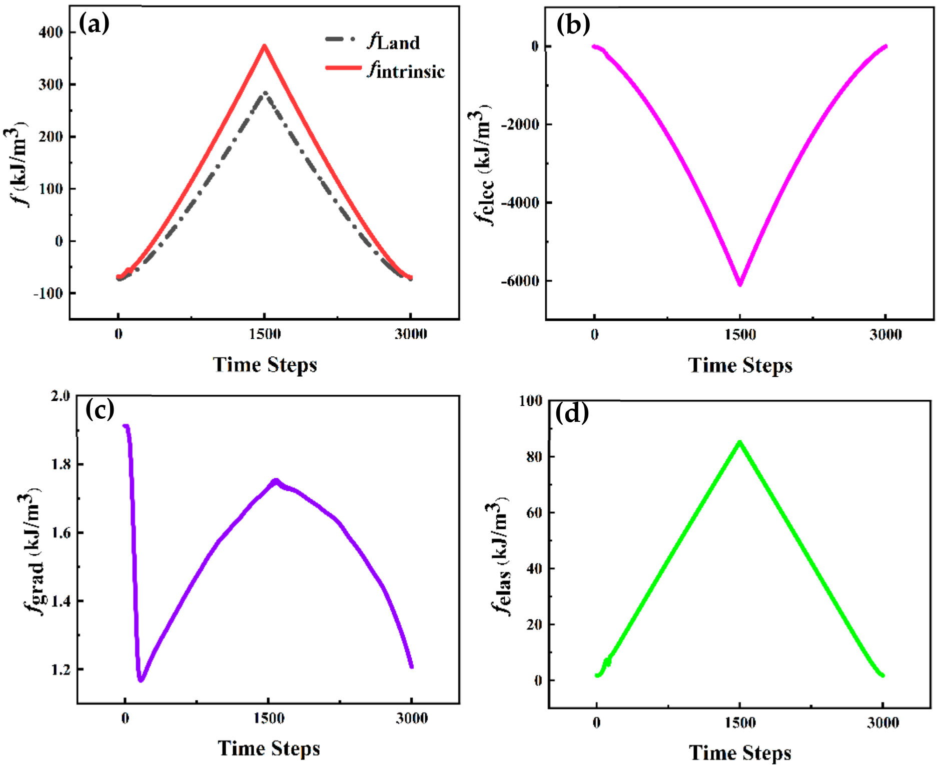

Figure S4. Dynamic temporal evolution of the volumetric average intrinsic energy density (consisting of Landau, gradient, and elastic energy density) and the average electrostatic energy density under different electrical conditions in $K_{0.75}Na_{0.25}NbO_3$ thin films. The 0–1500 time steps represent the loading process while the 1500–3000 represent the unloading process. (**a**) Landau and intrinsic energy density. **(b)**Electrostatic energy density. (**c**) Gradient energy density. (**d**) Elastic energy density.
